# Completion rates and psychosocial intervention effectiveness in an Australian substance use therapeutic community

**DOI:** 10.1186/s13011-018-0170-5

**Published:** 2018-09-24

**Authors:** Michael Harley, Sabrina Winona Pit, Trent Rees, Susan Thomas

**Affiliations:** 1 Lismore Base Hospital, Lismore, Australia; 2 Western Sydney University, University Centre for Rural Health, Sydney, Australia; 3 University of Sydney, University Centre for Rural Health, Sydney, Australia; 4 The Buttery, Bangalow, Australia; 5 Mental health and behavioural science, Graduate Medicine, School of Medicine, Faculty of Science, Medicine and Health, University of Wollongong, Wollongong, Australia; 6 61 Uralba Street, Lismore, NSW 2480 Australia

**Keywords:** Anxiety, Depression, Substance use treatment, Completion

## Abstract

**Background:**

Program attrition is a major problem in substance use treatment. It is not clear which client and treatment variables are related to successful completion. This study aimed to identify client variables associated with Therapeutic Community (TC) completion. A secondary aim was to investigate changes in entry and exit scores on psychosocial outcome measures.

**Methods:**

Retrospective quantitative analysis of data collected from 193 Australian TC residents, over 3.5 years. Variables measured included: demographics; Depression, Anxiety, Stress Score (DASS-21) and World Health Organisation Quality of Life 8 questions (WHOQOL-8).

**Results:**

Completion rates were 30.6%. High Money WHOQOL-8 scores, suggestive of minimal financial stressors, positively predicted completion. Multivariate analyses showed that negative predictors of completion were: amphetamine being primary substance of concern, aggression, high Relationship WHOQOL-8 scores, suggestive of positive relationships, and younger or older age. Those in the program demonstrated clinically significant psychological improvement and significant improvement in all quality of life scores over time. The degree of psychometric improvement was most pronounced in those who completed the course, with the exception of depression, stress, and money problems.

**Conclusion:**

The findings provide an understanding of specific predictors of program completion which may help to identify high-risk clients and inform program improvement. Early attrition rates may be reduced by monitoring and supporting high-risk clients. Overall, psychometric improvement occurred amongst both completers and non-completers overtime but is most prominent amongst course completers, with the exception of depression, stress, and money problems. Future research could potentially focus on amphetamine users and shortened TC programs, focusing on acute psychosocial intervention.

## Background

In Australia, substance use is conservatively estimated to cost $55 billion annually, in terms of lost productivity, health impacts, crime, road accidents and fires [1]. Internationally, 29 million people are estimated to have a Substance Use Disorder (SUD), resulting in over 200,000 deaths annually [2]. SUD is multidimensional, affecting the biological, psychological and social domains of individuals [3]. Treatment is generally long-term, multi-modal with repeated episodes of care, reflecting relapse-abstinence cycles [3].

Therapeutic Community (TC) programs are used in numerous countries around the world as a means to help individuals to maintain abstinence and enable social rehabilitation [4]. TCs share a common core of having the role of the treatment community as the primary agent of client change, however their designs are very heterogeneous [5]. This reflects the tailoring of programs to specific psycho-social sub types, thereby providing a more appropriate service [4]. A consequence of this heterogeneity is that studies of TCs are often unable to be compared, making overall evidence for programs weak [4]. However, they are considered an effective model of care [6, 7] and have demonstrated improved social rehabilitation outcomes [4], including: decreased substance use, lower rates of crime, higher rates of employment and higher levels of psychological functioning [4, 7].

Effectiveness of TCs can be measured in terms of initial and ongoing engagement with the program, completion of the program, short-term change in wellbeing and long-term outcomes in terms of functioning after program completion. Factors affecting completion rates of TCs are important for two reasons. Firstly, although TCs have been shown to be effective in the treatment of substance use disorders, completion rates are low, ranging from 9 to 56% [4]. Low completion rates also occur for other intervention types for SUD. This highlights the complexity of treating SUD, and suggests further research is required to improve completion rates. Secondly, the completion of a TC program is the strongest predictor of positive long-term outcomes [4].

There are several factors previously found to be positively associated with TC completion. A meta-analysis found that those who were relatively older were more likely to complete, however age groups varied across studies and mean ages of participants were relatively young, in the 20s to 30s [4]. Additionally, shorter duration TCs had higher completion rates [4]. Treatment variables generally have been found to be stronger positive predictors of completion than client variables [8]. Additionally, having previously completed a treatment for SUD positively predicts completion [9], as does good physical health [9]. Being currently in prison [9] or having a criminal justice referral [10, 11] positively predict completion. Additionally, having early positive responses to TC social processes was a positive predictor of completion [12]. TC members having high perceptions of orderliness and being in a well-regulated environment was a positive predictor of completion [13]. Another study found that the use of corrective feedback between peers to alert them to lapses from expected behaviour (“Pull-Ups”) was predictive of completion [14]. One study found that being White (versus coming from an ethnic minority) was a positive predictor of completion [11]. Factors found to be negatively associated with completion include recent prison release [9], low confidence in completion [9], impaired decision-making ability [15] and borderline personality disorder [16]. All other variables discussed in the literature have been found to have no significant association with TC completion. Of note, having a concurrent psychiatric disorder was not found to affect TC completion [4, 9, 17]. A 2012 systematic review found conflicting results regarding primary substance of use as a predictor of completion; and weak evidence suggesting severity of substance use as a negative predictor [4]. Self-reported impulsivity was also found to have no association with TC completion [18].

Psychosocial change in clients of TCs has been previously investigated by one systematic review and two additional studies [4, 19, 20]. These studies aimed to confirm long-term effectiveness of TC completers versus non-completers, or to compare long-term effectiveness of TC versus other rehabilitation modalities. A gap in the literature exists as to whether TCs improve short-term psychosocial outcomes of clients with SUD (as compared to the classic focus on long-term abstinence and reintegration into society). The relevance of this evidence gap is highlighted by a recent Australian TC study, which found psychiatric case management was indicated in 87% of clients on entry, with mean overall health scores two standard deviations below population norm, and Depression, Anxiety and Stress Scale (DASS) scores indicative of severe-extremely severe psychopathology in over 40% of clients [9].

‘The Buttery’ is a drug free, not-for-profit, charitable TC in Northern New South Wales, Australia. It provides residential substance use rehabilitation for male and female adults who are addicted to drugs, alcohol or both. Clients must be clean and free from drugs and alcohol for at least four days prior to joining the community. Its guiding philosophy is that addiction is not a consequence of choice, but rehabilitation is. The program is not affiliated with any religious or political group. It offers support in early recovery from substance use disorders of an understanding group of people through its TC, in a home-like rather than institutional-like environment.

It utilises a peer-based hierarchy, whereby clients take on additional group responsibilities as they progress through the program. Clients participate in evidence-based structured group and individual learning and therapy. The program consists of three phases, totalling seven months. Early withdrawal can be voluntary or involuntary. There is zero-tolerance to substance use relapse, which results in involuntary withdrawal. The service has a high demand, with waiting lists typically exceeding 12 months.

There is a need for further research examining client characteristics that predict completion rates, and short-term wellbeing outcomes for clients using the TC model of treatment. While TC approaches have local variability in content, their core approach of having a safe community as the primary therapeutic agent is consistent. Therefore, the systematic study of program outcomes in terms of completion rates and outcomes for clients of TCs will help to build knowledge regarding the efficacy of the TC model of treatment for improving the wellbeing of those with substance use disorders. Because substance use disorders affect the whole person, it is necessary to assess both psychopathology and multi-faceted quality of life to assess change associated with interventions. Additionally, it is likely that some client factors, for example, the main substance of concern, relationship factors, symptom profiles and socioeconomic factors may show common patterns across TCs in terms of predicting engagement and completion.

The primary aims of this study were to identify factors associated with TC completion as well as investigate the change measured in entry and exit psychometric data and quality of life (QOL). These outcomes will add to the body of evidence of TC completion. This information will also provide an understanding of specific predictors of program completion, facilitate identification of high-risk clients and inform program improvement.

A further aim was to investigate the psychosocial outcomes of clients with SUD. This will highlight any psychosocial differences in non-completers versus completers.

## Method

### Procedure

This study considered data routinely collected from TC clients admitted between January 2013 to July 2016. The data was collected via face-to-face interviews by TC staff and use of a computer based questionnaire upon program entry and exit. Consent for the data to be used in research was obtained and recorded during the admission interview. Ethical approval was granted by Western Sydney University (H11353) and University of Wollongong (NSA16/009) Ethics Committees.

### Measures

The primary outcome measure was course completion. This is defined as completion of the third phase of the program. This typically occurs at 217 days; however, clients can progress more slowly if program goals are not achieved. To ensure measures were clinically relevant to SUD treatment and met Australian standards; outcome measures were used from Network of Alcohol and Other Drugs Agencies (NADA) ‘Client Outcomes Management System’ [21]. This includes two validated psychometric assessment tools: 21-Item Depression, Anxiety, Stress Scale (DASS) [22] and World Health Organisation Quality of Life 8 questions (WHOQOL-8, also referred to as EUROHIS-QOL 8-Item Index) [21].

The DASS is a quantitative assessment tool which measures psychological distress in three domains: Depression, Anxiety and Stress. It has been validated for use in drug and alcohol services as well as across culture and age groups [21, 22]. It is noted that when interpreting DASS scores, a low score correlates to low morbidity. Further DASS has been validated to categorically correspond to clinical severity, with scores corresponding to clinical categories including Normal, Mild, Moderate, Severe and Very Severe, based on normative data [22].

WHOQOL-8 is a shortened adaptation of the WHOQOL-100, and has been validated for substance use and mental health disorders, cross cultural and Australian contexts [21]. It consists of eight questions regarding satisfaction with: ‘Health’, ‘Energy’, ‘Money’, ‘Daily Living’, ‘Self-Satisfaction’, ‘Relationships’, ‘Living Place’, and overall perception of QOL (‘Quality’) [21]. Questions are scored from 1 to 5, where high scores correlate to high satisfaction [21]. WHOQOL-8 scores do not categorically correspond to clinical severity; they are interpreted as relative change in perceived QOL [21].

Other NADA measures used include primary substance of use data and a categorical (Yes/No) questionnaire on other self-reported issues (other non-drug addictions, aggression issues, self-harm or suicidality issues, and risky behaviours). Additionally, rurality data was collected (residence prior to program commencement) as rural health is an Australian national health priority [23]. Rurality was measured using the Modified Monash Model, which is a geographical classification dependant on the distribution of medical services used by Australian Department of Health [24].

### Sample

Exclusion criteria were clients who did not consent to the research and clients who had not completed or exited the course at time of data analysis. A total of 257 clients were admitted to the program over this period, of which 44 did not give consent and a further 20 had not yet completed or exited the course; resulting in a sample size of 193. For a logistic regression a priory power analyses, assuming an odds ratio of 1.5, a power of 0.8, a probability of a completer prevalence of 0.3, alpha of 0.05 and a one tailed test, requires a sample size of 190.

### Statistical analysis

All data were analysed using SAS 9.3 (SAS Institute, Cary, NC, USA). Descriptive statistics were calculated for sample characteristics, psychopathology, quality of life and completion rates. This included before and after measures of psychopathology and quality of life, to gain an understanding of change in wellbeing from entering to exiting the program.

Chi squared tests were used to determine whether completers differed from non-completers in terms of important categorical demographic characteristics. Data were categorically grouped prior to univariate analysis to provide clinically meaningful outcomes. Age in years was categorised as: < 25, 26–50, > 51. DASS domains were categorised as: Normal-Mild, Moderate-Very Severe. Similarly, Primary Substance of Use was tested in a series of major substances (alcohol, amphetamines, cannabis, heroin) individually against ‘not using the substance’. Sub categories of the NADA self-reported issues were grouped so that a positive response to any sub category was recorded as a positive category response. T-tests were used to determine whether completers and non-completers differed in WHOQOL-8 scores as this tool is not validated for classification into categories of severity (21).

Variables that had an association with completion at *p* < 0.25 in univariate analyses were entered into a logistic regression model. Stepwise multivariate logistic regression was performed to determine odds ratios for factors associated with completion. For the multivariate modelling, DASS scores were considered as continuous variables to allow for maximum use of distribution information given the relatively small sample. Age was entered into the model as age and age*age to allow for the U-shape distribution to be modelled.

Repeated measures analysis of variance (RMAV), using PROC MIXED, was used to assess the psychometric measurements (DASS and WHOQOL-8 scores) over time (entry compared to exit) where the time by group interaction indicates a difference over time between completers and non-completers. An unstructured covariance model was used.

## Results

### Client characteristics

Client characteristics are presented by gender in Table 1. The overall mean age was 37.3 years (SD 9.24, range 20–66). Males accounted for 55.4% of clients. There were no notable differences in gender observed for the presented variables, except that men had higher proportions of risky behaviour (*P* = 0.038) and reported higher self-satisfaction scores (*P* = 0.0018) on program entry than women. Most clients were from urban areas with the highest level of access to health care (MMM1). The most common primary substance of use was alcohol, with amphetamines being second most common. The mean DASS scores upon admission were suggestive of Moderate Depression, Anxiety, and Stress levels. The most common other self-reported problems were risky behaviours, self-harm or suicidality issues and aggression.

**Table 1:** Client characteristic data (*N* = 193)

	Male		Female		Test-statistic	*P*-value
(*N* = 193)	(*n*)	(%)	(*n*)	(%)		
Gender	107	55.4	86	44.6		
Rurality						
MMM1	62	57.9	58	67.4	^1^Z = 1.2036	0.229
MMM2	5	4.7	3	3.5		
MMM3	12	112	7	8.1		
MMM4	14	13.1	8	9.3		
MMM5	14	13.1	9	10.5		
MMM6	0	0.0	1	1.16		
MMM7	0	0.0	0	0.0		
Primary drug						
Alcohol	51	48.6	44	53.0	^2^ χ(1) = 2.135	0.7110
Amphetamines	17	16.2	14	16.9		
Cannabis	12	11.4	6	7.2		
Heroin	17	16.9	10	12.1		
Other	8	7.6	9	10.8		
Self-reported problems						
Other addictions	13	12.2	11	12.8	^2^ χ(1) = 0.018	0.893
Self-Harm/Suicidality	62	57.9	51	59.3	^2^ χ(1) = 0.036	0.849
Aggression	49	45.8	42	48.8	^2^ χ(1) = 0.177	0.674
Risky Behaviour	94	87.9	65	76.5	^2^ χ(1) = 4.310	^4^ *0.038*
Gambling	13	12.15	8	9.4	^2^ χ(1) = 0.364	0.546
	(Mean)	(SD)	(Mean)	(SD)		
Age (years)	37.22	9.45	37.44	9.03	^3^T(191) = 0.16	0.871
DASS on Entry						
Depression	15.78	10.89	17.24	11.63	^3^T(183) = 0.88	0.378
Anxiety	12.55	8.34	13.04	9.48	^3^T(183) = 0.37	0.713
Stress	16.70	8.78	17.74	9.57	^3^T(183) = 0.77	0.441
WHOQOL-8 on Entry						
Quality	3.12	0.99	3.00	0.96	^3^T(190) = −0.86	0.393
Health	2.94	1.01	2.86	1.09	^3^T(190) = −0.56	0.576
Energy	3.26	1.05	3.04	1.03	^3^T(190) = −1.50	0.136
Money	2.98	1.32	3.09	1.14	^3^T(190) = 0.62	0.534
Daily Living	3.11	1.13	3.22	1.13	^3^T(190) = 0.68	0.497
Self-Satisfaction	2.70	0.92	2.26	1.00	^3^T(190) = −3.17	^4^ *0.0018*
Relationships	2.78	1.11	2.61	1.12	^3^T(190) = − 1.01	0.313
Living Place	3.02	1.24	3.16	1.15	^3^T(190) = 0.84	0.403

*MMM* = Modified Monash Model, a measure of location and medical service access. *MMM1* = Major cities, *MMM2* = Regional centres; *MMM3* = Large rural towns, *MMM4* = Medium rural towns, *MMM5* = Small rural towns, *MMM6* = Remote communities, *MMM7*  very remote communities

*DASS* = Depression, Anxiety, Stress Scale

*WHOQOL-8* = World Health Organisation Quality of Life 8 Questions

^1^Jonckheere-Terpstra Test; ^2^Chisquare test; ^3^T-test; ^4^ P<0.05

### Treatment retention

Treatment retention data are presented in Table 2. Course completion was achieved for 30.6% of clients. The median length of stay was 112 days (SD 78.5, range 1–247). Voluntary early exit occurred in 51.8% of cases and 17.6% were involuntarily removed from the program due to failure to maintain abstinence or repeatedly not meeting the requirements of the program. The greatest exit rate (combined voluntary and involuntary exits) occurred within the first 14 days of the program commencement, with 10.4% of clients exiting over this period. The second highest exit rate occurred between weeks 5–8, with 3.6% leaving weekly over this period (14.6% over four weeks).

**Table 2:** Treatment retention data

	*N* = 193	
	(*n*)	(%)
Completion status group		
Completers	59	30.6
Non-completers	134	69.4
Non-completer exit status		
Voluntary	100	51.8
Involuntary	34	17.6
Non-completer duration of stay		
Weeks 1–2	20	10.4
Weeks 3–4	9	4.7
Weeks 5–6	14	7.3
Weeks 7–8	14	7.3
Weeks 9–10	5	2.6
Weeks 11–12	11	5.7
> 12 Weeks	61	31.6
Median length of stay (days)	112 (SD 78.5, Range 1–247)	

### Differences between completers and non-completers

Data showing differences between completers and non-completers are presented in Table 3. Clients at both age extremes were a minority in numbers and less likely to complete the course (*P* = 0.026). Those less than 25 years (*n* = 16) only had an 11.19% completion rate; those greater than 50 years (*n* = 18) had an 11.19% completion rate. Non-completers had a higher incidence of self-reported aggression problems (*p* = 0.006), self-reported current or previous self-harm or suicidality issues (*P* = 0.017), and lower Money WHOQOL-8 score (*P* = 0.011) on entry. Whilst not achieving statistical significance, non-completers also had higher rates of categorical (*P* = 0.062) and mean Depression (*P* = 0.065) on entry, were more likely to come from rural areas with reduced health care access (MMM2–7; *P* = 0.087) and have amphetamine as the primary substance used (*P* = 0.052).

**Table 3:** Differences between completers and non-completer: univariate analyses

	Total		Completers	Non-Completers	Test-statistic	*P*-value
	*N* = 193		*N* = 59	*N* = 134		
	*n*	%	%	%		
Gender					^1^χ(1) = 0.050	0.823
Male	107	55.44	54.24	55.97		
Female	86	44.56	45.76	44.03		
Age					^1^χ(2) = 7.309	^4^ *0.026*
< 25 yr	16	8.29	1.69	11.19		
26–50 yr	159	82.38	93.22	77.61		
> 51 yr	18	9.33	5.08	11.19		
Mean (sd) Age (years)			36.86 (6.99)	37.52 (10.09)	^2^T(156) = 0.52	0.603
Rurality					^1^χ(1) = 2.934	0.087
MMM 1 (Metro)	120	62.18	71.19	58.21		
MMM 2–6 (Non-Metro)	73	37.81	28.81	41.79		
Depression on Entry (DASS)					^1^χ(1) = 3.478	0.062
Normal-Mild	85	45.95	55.93	41.27		
Moderate-Very Severe	100	54.05	44.07	58.73		
Mean (sd)			14.20 (10.80)	17.46 (11.30)	^2^T(183) = 1.86	0.065
Anxiety on Entry (DASS)					^1^χ(1) = 0.003	0.958
Normal-Mild	81	43.78	44.07	43.65		
Moderate-Very Severe	104	56.22	55.93	55.93		
Mean (sd)			11.61 (8.31)	13.31 (9.06)	^2^T(183) = 1.22	0.224
Stress on Entry (DASS)					^1^χ(1) = 0.218	0.641
Normal-Mild	102	55.14	57.63	53.97		
Moderate-Very Severe	83	44.86	42.37	46.03		
Mean (sd)			15.61 (8.41)	17.89 (9.38)	^2^T(183) = 1.59	0.114
Primary Substance^3^						
Alcohol	95	50.53	56.90	47.69	^1^χ(1) = 1.359	0.243
Amphetamines	31	16.49	8.62	20.0	^1^χ(1) = 3.771	0.052
Cannabis	18	9.57	6.90	10.77	^1^χ(1) = 0.695	0.405
Heroin	27	14.36	17.24	13.08	^1^χ(1) = 0.566	0.452
Other Drugs	17	9.04	10.34	8.46	^1^χ(1) = 0.173	0.678
Self-Reported Problems						
Non-Drug Addictions	39	20.21	20.34	20.15	^1^χ(1) = 0.0009	0.976
Aggression	91	47.15	32.20	53.73	^1^χ(1) = 7.619	^4^ *0.006*
Self-Harm/Suicidality	113	58.55	45.76	64.18	^1^χ(1) = 5.725	^4^ *0.017*
Risk Taking Behaviours	159	82.81	84.75	81.95	^1^χ(1) = 0.2237	0.636
WHOQOL-8 on Entry	Mean (sd)		Mean (sd)	Mean (sd)		
Quality	3.07 (0.99)		3.10 (0.98)	3.05 (0.98)	^2^T(190) = −0.32	0.749
Health	2.98 (1.04)		2.91 (1.04)	2.90 (1.05)	^2^T(190) = −0.08	0.937
Energy	3.16 (1.05)		3.27 (1.11)	3.11 (1.01)	^2^T(190) = −0.97	0.333
Money	3.01 (1.24)		3.37 (1.29)	2.88 (1.20)	^2^T(190) = −2.57	^4^ *0.011*
Daily Living	3.17 (1.13)		3.15 (1.20)	3.17 (1.10)	^2^T(190) = 0.07	0.942
Self-Satisfaction	2.50 (1.00)		2.52 (1.06)	2.50 (0.95)	^2^T(190) = −0.19	0.850
Relationships	2.67 (1.12)		1.56 (1.15)	1.77 (1.10)	^2^T(190) = 1.19	0.236
Living Place	3.07 (1.19)		3.20 (1.16)	3.03 (1.22)	^2^T(190) = −0.92	0.357

*MMM* = Modified Monash Model, a measure of location and medical service access, *DASS* = Depression, Anxiety, Stress Scale; WHOQOL-8 = World Health Organisation Quality of Life 8 Questions

^1^Chisquare test; ^2^T-test; ^3^*N* = 188, comparison made: primary substance us other primary drug substance; ^4^*P*<0.05

### Relative strength of variables predicting completion

Results from the multivariate logistic regression predicting completion are presented in Table 4. Higher Money (WHOQOL-8) score on entry positively predicted completion OR = 1.57 (95% Confidence Intervals (CI): 1.14, 2.16). Negative predictors, in order of strength, were amphetamine as primary substance of concern, OR = 0.29 (95% CI: 0.01, 0.89), aggression problems, OR = 0.41 (95% CI: 0.19, 0.86), and higher Relationship ratings on entry, (WHOQOL-8) OR = 0.60 (95% CI, 0.41, 0.86). Age had a non-linear relationship with completion, with the curve being parabolic. The peak association with completion was at 38 years with the odds ratio declining steadily towards negative associations with completion in the younger and older age groups.

**Table 4:** Multivariate stepwise logistic regression series of associations with completion

Predictors of completion	Odds ratio	95% CI	*P*-value
N = 193			
Money Ratings (WHOQOL-8)	1.57	(1.14, 2.16)	^1^ *0.005*
Amphetamines as Primary Substance	0.29	(0.01, 0.89)	^1^ *0.030*
Aggression Problems	0.41	(0.19, 0.86)	^1^ *0.017*
Good Relationship Ratings (WHOQOL-8)	0.60	(0.41, 0.86)	^1^ *0.007*
Self-Harm/Suicidality Problems	0.52	(0.25, 1.07)	0.074
Depression on Entry (DASS)	0.97	(0.94, 1.01)	0.193
Age	–	–	^1^ *0.010*
Age* Age	–	–	^1^ *0.007*

*DASS* = Depression, Anxiety, Stress Scale

*WHOQOL-8* = World Health Organisation Quality of Life 8 Questions

^1^*P*<0.05

### Psychosocial outcome measures

Table 5 shows the comparison between completers and non-completers, the change in DASS and WHOQOL-8 from entry to exit and interactions between group (completers versus non completers) and time. Improvements occurred in the estimated mean values of all psychometric domains over time (time effect, *P* < 0.001). Overall, the completers had better psychometric ratings than non-completers (completion effect) with the most notable differences found for Money (*P* < .001). The completion*group effect demonstrated that the improvement of psychometric outcome measures was greater in those who completed the course, with the largest completion*time effect being for improved relationship ratings for those who completed the course compared to non-completers (*P* < .001). However, no significant differences were found for Depression (*P* = 0.230), Stress (*P* = 0.098), and Money (*P* = 0.343) when comparing the change scores from entry to exit between completers and non-completers.

**Table 5:** Comparison between completers and non-completers (group) on change in DASS and WHOQOL-8 from entry to exit (time)

	Completers				Non-completers					Time effect			Completion effect			Time*completion effect		
	Entry		Exit		Entry		Exit			DF	F	P	DF	F	P	DF	F	P
	Estimate	SE	Estimate	SE	Estimate	SE	Estimate		SE									
DASS																		
Depression	14.20	1.47	4.83	1.12	17.41	1.08	10.28		0.83	166	79.3	^1^ *<.0001*	166	10.59	^1^ *0.001*	166	1.45	0.230
Anxiety	11.61	1.14	4.07	0.95	13.06	0.84	8.36		0.70	166	80.1	^1^ *<.0001*	166	6.69	^1^ *0.011*	166	4.33	^1^ *0.039*
Stress	15.61	1.19	7.93	1.08	17.39	0.88	12.34		0.80	166	64.7	^1^ *<.0001*	166	6.96	^1^ *0.009*	166	2.77	0.098
WHOQOL-8**																		
Quality	3.10	0.13	4.27	0.11	3.05	0.09	3.74		0.08	171	101	^1^ *<.0001*	171	6.380	^1^ *0.0125*	171	6.9	^1^ *0.009*
Health	2.92	0.14	4.00	0.11	2.94	0.10	3.63		0.08	171	80.6	^1^ *<.0001*	171	2.14	0.146	171	3.91	^1^ *0.050*
Energy	3.27	0.14	4.25	0.12	3.10	0.10	3.69		0.08	171	67.3	^1^ *<.0001*	171	9.1	^1^ *0.003*	171	4.03	^1^ *0.046*
Money	3.37	0.16	4.22	0.14	2.82	0.11	3.45		0.10	171	42.5	^1^ *<.0001*	171	21.14	^1^ *<.0001*	171	0.91	0.343
Daily Living	3.15	0.15	4.47	0.11	3.18	0.11	3.81		0.08	171	102	^1^ *<.0001*	171	6.28	^1^ *0.013*	171	12.7	^1^ *0.001*
Self-Satisfaction	2.53	0.13	4.19	0.12	2.51	0.09	3.61		0.09	171	231	^1^ *<.0001*	171	5.66	^1^ *0.018*	171	9.67	^1^ *0.002*
Relationships	2.56	0.15	4.05	0.12	2.76	0.11	3.40		0.08	171	127	^1^ *<.0001*	171	2.76	0.099	171	20.3	^1^ *<.0001*
Living Place	3.20	0.15	4.20	0.12	3.08	0.11	3.63		0.08	171	56.4	^1^ *<.0001*	171	6.89	^1^ *0.009*	171	4.68	^1^ *0.032*
Note: 25 Non-completers did not complete all components of the exit interview, resulting in reduced N values																		
DASS categorical																		
	Normal	Mild	Moderate	Severe	Very Severe													
Depression	0-9	10-13	14-20	21-27	28-42													
Anxiety	0-7	8-9	10-14	15-19	20-42													
Stress	0-14	15-18	15-25	20-33	34-42													

^1^*P* <0.05

Table 5 also shows that within the DASS domains, Depression scores lowered over time for both groups, indicating a clinical categorical change from Moderate to Normal [22]. Anxiety also improved over time from Moderate at entry (11.61) to Normal on exit (4.07) for completers and from Moderate on entry (13.06) to Mild on exit (8.36) for non-completers. Stress scores improved from Mild to Normal over time for both completers and non-completers.

Within the QOL domains, Money showed the greatest initial difference between the two groups (*P* < .0001). The effect of time was also significant, but the group*time interaction was not significant, suggesting that money concerns improved across both groups overtime.

## Discussion

### Statement of principal findings

Around one third of participants completed the program. Multivariate logistic regression demonstrated that those who reported higher satisfaction levels in relation to money had higher odds of program completion. Additionally, those who previously primarily used amphetamines, self-reported aggression problems, were more satisfied with their relationships and those who were at the extreme ends of age in the sample (younger or older) had lower odds of program completion. Further, our study showed that the TC program improved psychological and quality of life scores among residents, as measured by clinically significant clinical improvements in DASS and WHOQOL-8 on exit irrespective of program completion. The degree of psychometric improvement was most pronounced in those who completed the course, with the exception of depression, stress and money ratings. This suggests that the improvements in ratings for these factors did not differ significantly between completers and non-completers.

### Relation to other studies

Previous studies have demonstrated mental health and QOL improvement during follow up post-TC attendance [4, 19, 20]. This study’s results in psychosocial improvement are consistent with the existing evidence. We additionally addressed a gap in previous literature by measuring short-term outcomes in both treatment completers and those who left the program prior to completion. Our results identified a clinically significant psychosocial therapeutic effect from TC program attendance, which is independent of completion. This extends the existing evidence regarding TC program outcomes. TC duration varies considerably, and higher completion rates are found for shorter programs [4]. It is possible that some programs are longer than necessary. If this is the case, individuals leaving before completion may be counted as having terminated prematurely, however they may have had sufficient benefits from the program to achieve improvement in mental health and quality of life. Further research should investigate the potential to broaden the scope of drug and alcohol TCs from chronic SUD rehabilitation into acute or shorter-term SUD intervention functions. The length of time of TCs varies greatly and may be somewhat arbitrary.

This study provided additional insight into some other complex areas. While the attrition rate is lower beyond 12 weeks, there is still a large (30%) exit rate after 12 weeks but prior to program completion. These figures are similar to other studies. This delayed attrition occurred despite clients successfully overcoming initial withdrawal symptoms and early adjustment issues. It may be that the delayed attrition in the program is due to social stressors, such as employment and finances or separation from positive relationships (partner or children). Indeed, those who reported higher levels of satisfaction with relationships upon entry were less likely to complete the program. It may be that clients with delayed attrition differ from individuals with early attrition in other important ways. Future studies are needed to investigate whether this is the case.

The results are consistent with previous research suggesting that individuals were more likely to leave treatment prematurely if their primary drug of concern was a stimulant [4]. The associations with completion found in our study did differ from some literature, as we found that both younger and older age predicted early treatment cessation, whereas a previous meta-analysis indicated that older age predicted premature departure [4]. This is partly expected due to variations in study parameters, settings and participants but may also reflect the known variation in TC program design. Additionally, we identified novel information about relationship and financial satisfaction as predictors of program completion which has not been identified in previous studies.

### Implications of the study

High attrition rates are expected in the first few weeks as SUD clients experience withdrawal effects and adjustment issues, and the current completion rate results are consistent with findings in previous TC studies [4]. The predictors of completion can be used to improve client recruitment for specific programs, with the aim of matching the type of clients offered intake to programs with the type of program where they have a higher likelihood of completion. Additionally, they could be used to better tailor interventions to meet clients’ individual needs by identifying at early stages those at higher risk of attrition and facilitating additional monitoring and support. An ideal situation would be for all TCs in a geographical region to assess and publish their individual predictors of completion. This may facilitate a centralised intake system to best match clients to TC programs based on client and TC program characteristics. The importance of this is highlighted by previous studies into TC completion predictors, which tend to find variation in predictors by TC program design [4]. Furthermore, the Buttery has a long waiting time of around a year to entry, which could be a hindrance to the recovery process. It may be that a system of prioritisation could be introduced to allow for a shorter course to be offered to high-risk individuals which would allow for more people to be served.

The delayed attrition of clients, in which 30% prematurely exited the program after three months, is likely to be due to broader mental health issues or social factors, as these clients overcame the initial withdrawal and adjustment issues. Our results suggest program improvements could include financial counselling services. Clients with financial stress (low Money satisfaction in WHOQOL-8) were less likely to complete the program, as demonstrated in multivariate analyses. Further, Money was the only WHOQOL domain that did not show improvement in completers compared to non-completers over time.

High Relationship satisfaction (WHOQOL-8) on entry was negatively associated with completion, presumably reflecting distress with separation from loved ones. Improvement to support clients with existing relationships external to the course, such as partners and other family, may be warranted. TCs require clients to have very restricted contact with people beyond the TC as part of the recovery process, to separate from existing patterns and habits and to focus on personal recovery and growth. A desire for contact with significant others beyond the program may contribute to early departure in some clients with strong bonds. Indeed, a recently completed qualitative study investigated reasons for early withdrawal from the TC program through semi-structured interviews with residents in 2017–2018. A recurring theme for early withdrawal was residents’ relationships [25]. It is also noted that Relationship ratings showed the second greatest QOL improvement in completers; presumably reflecting a group with poor pre-course relationships who develop positive social skills and friends throughout the program. This indicates that the TC may be particularly filling a need for those who are initially lacking close relationships and friendships.

Our assessment of short-term outcomes identified that non-completers may show significant improvements in wellbeing even though they did not complete the entire program. The introduction of a short SUD interventional program, conducted in addition to the existing course could be considered. The short course could be utilised in combination with pre-admission screening, whereby those at higher risk of non-completion attend the short course as an introduction or acute intervention. This could further improve the psychological and social wellbeing for those unable or unwilling to complete the course, and potentially also lead to higher completion rates of the full course. The concept of a short interventional program is also supported by existing evidence, as higher completion rates and improved long-term outcomes are found in shorter TC programs [4]. Previous TC completion is also associated with a higher completion rate [9]. Nemes et al. found improved outcomes with a short period of inpatient treatment followed by outpatient intervention [26]. There is also a potential to address negative perceptions of non-completion, which have been found to be associated with reduced completion rates [9].

The lower completion rates in those clients at the younger and older ends of our sample may indicate that they did not feel the same levels of peer support or belonging in the TC, possibly due to being of a different age to others in the group. Further, qualitative, research is warranted to determine if this is the case.

### Limitations of the study

The relatively small sample size limited the ability to compare several subgroups against completion. In univariate analyses only amphetamine was trending towards being significantly different (*P* = 0.052) so only this primary drug group was included in the multivariate analyses. It is important to note that while the numbers are small for amphetamine relative to other drugs of concern at present, the use of amphetamine is a currently increasing problem [27]. Early program withdrawal may be related to withdrawal effects, such as paranoia, irritability or inability to regulate emotions, which in turn may lengthen the time taken see positive effects and increase the likelihood of premature departure. More research is needed to explore this area. Indeed, an inquiry into crystal methamphetamine use reported that there is lack of research into amphetamine being used as the primary drug of concern, which restricts the development of services to address the needs of this growing group [27]. Missing data resulted in a reduced sample size for parts of the analyses. Nine clients were missing either entry DASS or WHOQOL-8 data, which likely had minimal impact. However, 25 clients (all non-completers) were missing exit DASS and WHOQOL-8 data. It is likely that those who were lost to follow up departed the program abruptly and had lower DASS and WHOQOL-8 scores; suggesting our findings are conservative. All non-demographic data relied on self-reported measures. Two clients reported entry DASS totalling < 2. This very low score is unlikely to be consistent with an objective assessment of a person with SUD requiring residential rehabilitation; however, could represent mood or insight-related psychopathology associated with SUD. As the measures have been validated for use in Australian Drug and Alcohol services (21), it is assumed this self-reporting bias is normalised across similar studies and as such had a negligible effect. Another study limitation was the number of variables that were compared, which could have increased the likelihood of type 1 errors.

### Future research

Qualitative studies into the causes of early exit are rare. One qualitative study has been conducted [28], however it was conducted in a niche population of judicial-referred paediatric clients. Quantitative predictors of completion studies within various TCs will enable improved matching of clients to TC program designs. This also relies on development to centralise and communicate these findings. Further research is needed to determine the relationship between length of stay and acute intervention effectiveness to determine the optimal duration suitable for conducting shortened interventions. A potential area of further research is to identify trends and completion rates for amphetamine users and possible mechanisms and reasons why amphetamine users are potentially less likely to complete programs, with a view to better meeting their needs.

## Conclusion

In conclusion, those who reported higher satisfaction levels in relation to money had higher odds of program completion. Those who used amphetamines as primary substance, self-reported aggression, those who reported positive relationships on entry into the program and those who were at the younger or older extremes of the sample had lower odds of program completion. Additionally, our study showed that the TC program improved psychological and quality of life scores overtime. These were clinically significant clinical improvements. The degree of psychometric improvement was most pronounced in those who completed the course, with the exception of depression, stress, and money problems. Early attrition rates may be reduced by improved recruitment, monitoring and supporting high-risk clients. Future research could potentially focus on amphetamine users and shortened TC programs, focusing on acute psychosocial intervention.

## Abbreviations

CI: Confidence Interval
DASS: Depression, Anxiety, Stress Scale
EUROHIS-QOL: European Health Interview Survey-Quality of Life
MMM: Modified Monash Model
NADA: Network of Alcohol and Other Drugs Agencies
QOL: Quality of Life
SD: Standard Deviation
SUD: Substance Use Disorder
TC: Therapeutic communities
USA: United States of America
WHOQOL: World Health Organisation Quality of Life
